# Rapid development of an iatrogenic aortic dissection following transcatheter aortic valve implantation

**DOI:** 10.1007/s12024-020-00219-2

**Published:** 2020-03-14

**Authors:** Julian Geile, Elke Doberentz, Burkhard Madea

**Affiliations:** Institute of Legal Medicine, University Hospital Bonn, Stiftsplatz 12, 53111 Bonn, Germany

**Keywords:** Transcatheter aortic valve implantation, Cardiac catheterization, Aortic valve stenosis, Complications, Dissection

## Abstract

Transcatheter aortic valve implantation (TAVI) represents a catheter-based and minimally invasive replacement of the aortic valve. TAVI is considered to be a relatively safe procedure and has evolved to a standard procedure in inoperable and high-risk patients with aortic stenosis. We present a case of an octogenarian who died in hospital less than a day after an initially satisfactory TAVI. Cardiologists suspected a combination of cardiogenic and hemorrhagic shock as the cause of death. Autopsy showed rupture of an extensive aortic dissection, which had developed within 24 h after the procedure. The cause of death was eventually defined as internal bleeding due to a rapid two-stage vascular process. The manner of death was considered accidental because an iatrogenic vessel injury beyond the aortic arch was causative for the death. This unusual case highlights the potential for rare, but fatal, complications within the scope of cardiac catheterizations, such as TAVI. Additionally, our findings suggest that these complications need to be recognized in the diagnostic process and management of post-interventional complications.

## Introduction

Aortic stenosis (AS) is one of the most prevalent form of cardiovascular disease in the Western world [1]. Grading of AS is commonly based on echocardiographic parameters (Table 1)*,* but these parameters may be discordant in a certain proportion of patients with severe AS [2]*.* Among other options, AS can be treated by cardiac surgery or catheter-based interventions. Transcatheter aortic valve implantation (TAVI) represents catheter-based and minimally invasive replacement of the aortic valve. TAVI has evolved to a standard procedure in inoperable and high-risk patients with AS. The first TAVI procedure in humans was performed in 2002 in France by Cribier [3] and over the years, more than 500,000 of these procedures were performed in more than 70 countries [4]. In Germany, more than 100,000 TAVI procedures have been performed since 2008 [5], and currently, more than 12,000 procedures are performed each year [6]. In the USA in 2016, a total of 54,782 TAVI procedures were reported to the Transcatheter Valve Therapy Registry [7]. With regard to potential complications, local vascular events are the most common complications after cardiac catheterizations, such as TAVI. Hemorrhage and hematoma usually emerge within 12 h after this procedure. In contrast, pseudoaneurysms may take days or weeks to become apparent [8] and they are reported to occur in 1.6% of TAVI interventions in which the ProGlide® System is used [9]. The risk of an iatrogenic aortic dissection (IAD) in TAVI ranges between 0.2% and 0.3% [10, 11] and may be caused by manipulations of the guidewire/delivery system or repositioning, retrieval, and/or retraction of the valve [12]. In these cases, dissection commonly occurs in the aortic root, but the ascending aorta may also be injured by catheters or guidewires [13].

**Table 1:** Echocardiographic grading of AS severity (modified from Baumgartner et al. 2017 [2])

Normal	Peak velocity (m/s)	Mean Gradient (mmHg)	Aortic Valve Area (cm^2^)
Mild	2.6–2.9	< 20	> 1.5
Moderate	3.0–4.0	20–40	1.0–1.5
Severe	≥ 4.0	≥ 40	< 1.0

We present a case of an octogenarian who died in the hospital within 24 h after an initially satisfactory TAVI because of rupture of a rapidly developed aortic dissection.

## Case report

**Fig. 1:**
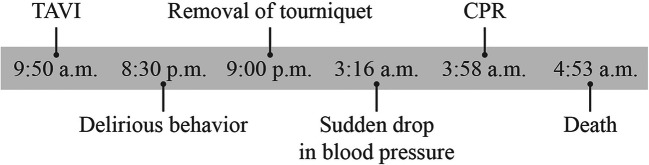
Timeline of crucial events

An 89-year-old man with severe symptomatic AS underwent elective TAVI. The patient’s demographics and the pre-interventional echocardiographic parameters are shown in Table 2. The procedure occurred via right transfemoral access (14 French) using the ProGlide® System (Abbott, Lake Bluff, Illinois, USA). A heart catheterization report stated that a stenotic aortic valve was passed with a Terumo Glidewire® (Terumo Corporation, Tokyo, Japan) and then changed to a Confida Wire™ (Medtronic, Dublin, Ireland). Initially, the prosthetic aortic valve (Medtronic CoreValve® Evolut™ R 29 mm) could not pass the aortic arch over this wire. Therefore, the cardiologist switched to a Lunderquist® Extra-Stiff Wire Guide (Cook Medical, Bloomington, Indiana, USA). This report did not state any explanation for why the valve could not pass the aortic arch using the first wire. Afterwards, the implantation proceeded without further complications and the prosthetic valve could have been implanted properly with a satisfactory result (Table 3). The patient was then admitted to the Intensive Care Unit in a stable circulatory condition. While the patient was in the Intensive Care Unit, he showed delirious behavior and removed his tourniquet at the groin, which resulted in an extensive hematoma at the puncture site. However, his hemoglobin level initially remained stable. At night, a sudden drop in blood pressure occurred and cardiopulmonary resuscitation (CPR) had to be performed. Ultrasound investigations of the heart and thorax ruled out pericardial effusion and free abdominal fluid, and the position of the valve prosthesis was described as regular. Only a small amount of pleural effusion in the left thoracic cavity was apparent. The hematoma at the left groin was described as identical to previous findings. Computed tomography was planned, but could not be performed because of rapid deterioration of the patient’s condition and initiation of CPR. A rapid decline in hemoglobin level was later observed and extensive CPR had to be initiated. The patient was eventually declared dead after 50 min of unsuccessful CPR. The physicians suspected a combination of cardiogenic and hemorrhagic shock as the cause of death and reported an uncertain manner of death. A timeline of the crucial events is shown in Fig. 1.

**Table 2:** Patients’ demographics and pre-interventional echocardiographic parameters

Sex	male
Age	89 years
BMI	22.6 kg/m^2^
Logistic EuroSCORE	23.23%
NYHA functional class	3
Peak aortic velocity	3.7 m/s
Mean aortic gradient	34 mmHg
Aortic valve area	0.8 cm^2^
Ejection Fraction	55%
Aortic regurgitation	mild
Mitral regurgitation	mild

**Table 3:** Left ventricular and aortic valve pressure before and after TAVI

	Before TAVI	After TAVI
Left ventricle (mmHg)	210/19	143/19
Aortic valve (mmHg)	169/75	123/61

**Fig. 2:**
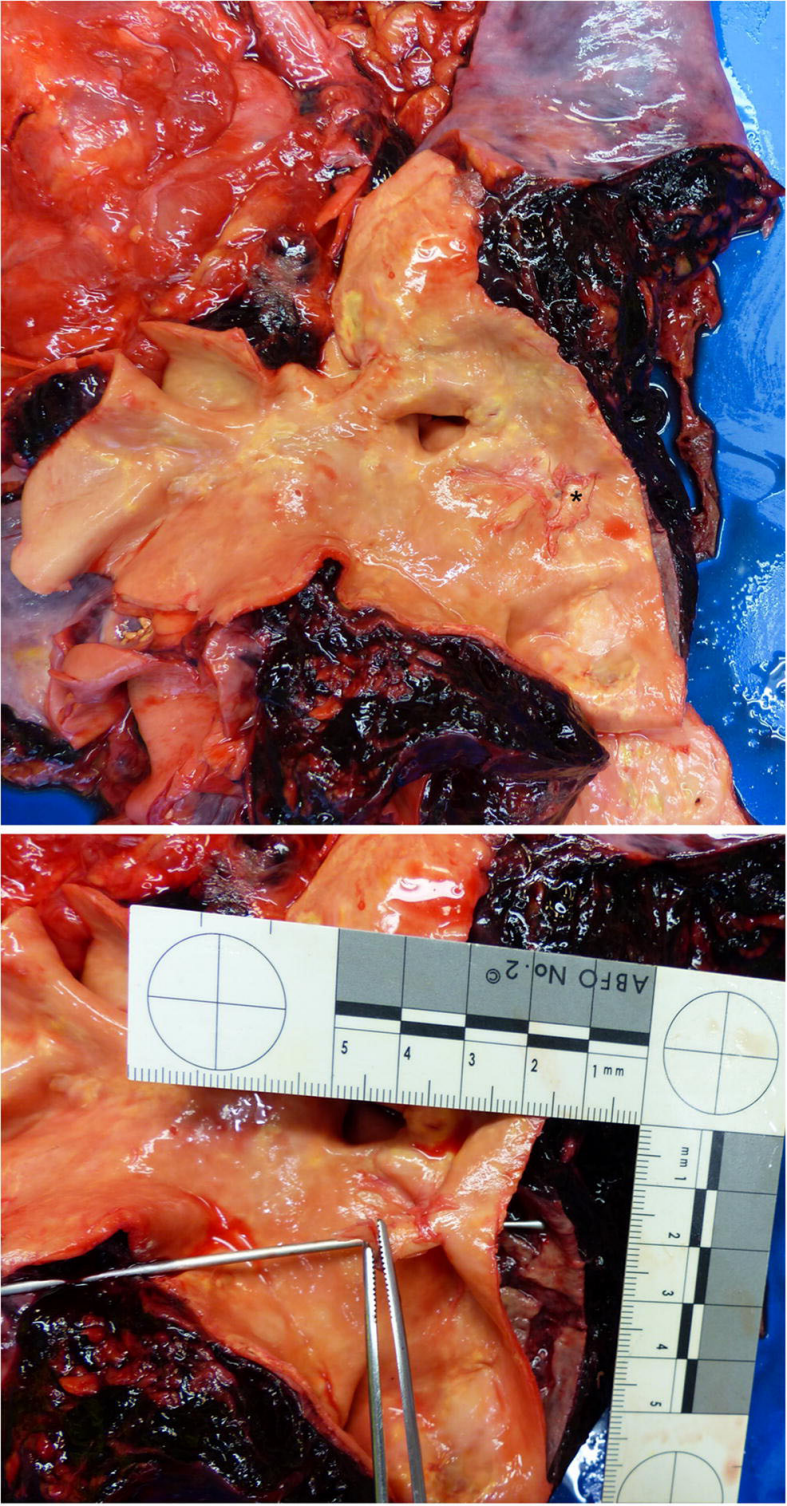
Aortic arch with a tear (*) after removal of the neck organs with surrounding hemorrhage

**Fig. 3:**
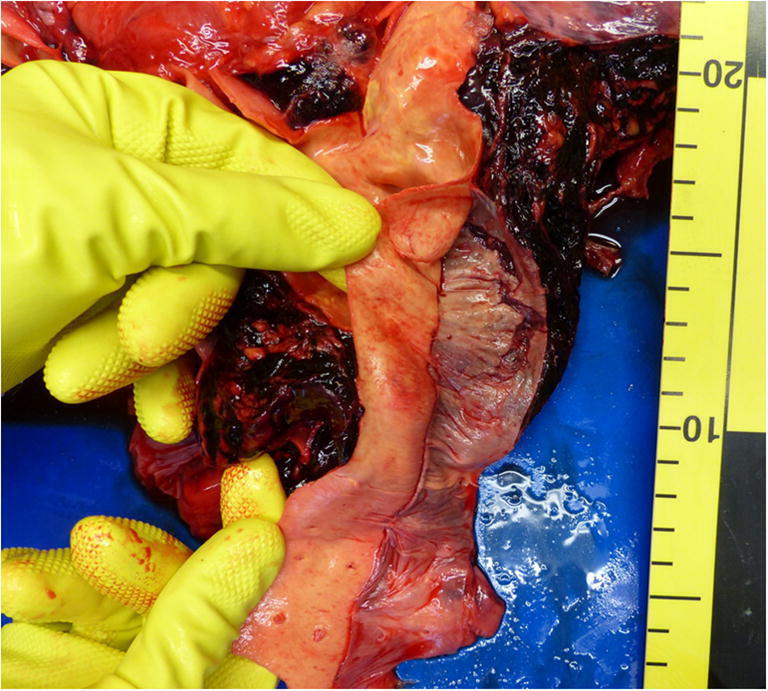
Upper segment of the 32 cm long aortic dissection

**Fig. 4:**
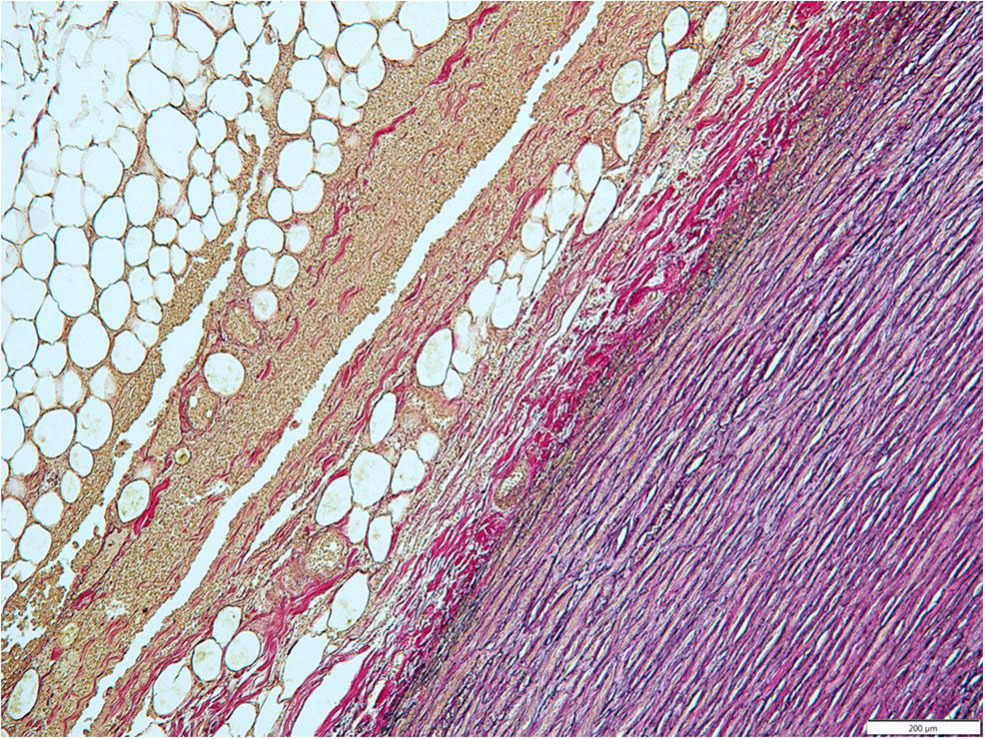
Elastica van Gieson stain. Dissection in the adventitia-media border with a predominantly regular layered pattern of elastic fibers in the tunica media (×100)

An autopsy showed a sparse livor mortis formation. The hematoma at the groin measured approximately 33 × 30 cm and stretched to the left renal capsule, but there was no blood or free fluid in the abdominal cavity. An aortic tear (approximately 0.8 × 0.4 cm) with extensive surrounding hemorrhage was located below the aortic arch (Fig. 2). Proceeding from this tear, a 32 cm long dissection in the thoracic and abdominal aorta wall had developed (Fig. 3). Approximately 800 ml of partial fluid and partially clotted blood were found in the left thoracic cavity. The prosthetic aortic valve appeared to be properly implanted. Histological examination of the aorta showed a dissection in the adventitia-media border (Fig. 4).

The cause of death was eventually defined as internal bleeding due to a two-stage vascular process that occurred within less than 24 h. The manner of death was defined as accidental because the iatrogenic vessel injury beyond the aortic arch was causative for the death. On the basis of the severity of the autopsy findings and the rapid progression, we could not confirm with absolute certainty that instantaneous treatment of the dissection could have prevented the death of the patient or that the intervention was executed improperly. Therefore, medical malpractice was ruled out from the viewpoint of forensic medicine.

## Discussion

TAVI represents an established and relatively safe alternative for inoperable and high-risk patients with AS stenosis. Despite this fact, the present case may be a helpful example to draw attention to a rare, but potentially fatal, complication within the scope of this procedure. Because of the constantly increasing numbers of TAVIs being performed, a considerable number of patients are likely to be affected by fatal vascular complications, such as IAD. Although the risk for IAD is relatively low, between 24 and 36 patients in Germany and between 109 and 164 patients in the USA could be affected by this life-threatening complication each year, considering the particular number of annual TAVI procedures.

On the basis of the provided medical records for the current case, we conclude that the aortic arch was damaged during the first unsuccessful attempt to position the prosthetic aortic valve. This event resulted in development of an extensive dissection, which eventually ruptured within less than 24 h after the intervention. The course of the clinical diagnostics was presumably distracted by the fact that the patient removed his tourniquet, which resulted in an extensive groin hematoma. In spite of this event, the cardiologists focused on this hematoma because local vascular bleeding represents the most frequent complications following cardiac catheterization. With the benefit of hindsight and considering that pleural effusion due to congestive heart failure is commonly bilateral [14], the small left-sided pleural effusion might have been mistaken for developing hemothorax as a result of rupture of the dissection. The medical records did not state if a mechanical chest compression device was used during CPR. However, mechanical force due to prolonged chest compression might have accelerated development and/or triggered rupture of the dissection. Nevertheless, we concluded that the dissection originated from the iatrogenic aortic injury because the aorta did not show any relevant inflammatory or degenerative alterations that might have led to development of a non-traumatic aneurysm.

Several case reports on IAD following TAVI have been published in which the dissection occurred either during the procedure [15, 16] or several days or even years later [17–20]. In those delayed cases, there was either endocarditis of the prosthetic valve or the dissection was located at the aortic annulus, the aortic root, or the valve cage area [17–19, 21]. In one case, a patient died during the intervention because of aortic arch rupture, but unfortunately no autopsy findings were presented [22]. Aminian et al. [23] reported a case of an octogenarian who died soon after valve implantation because of a 1 cm large perforation of the descending thoracic aorta. This resulted in a large, left-sided hemothorax, but the exact time frame was not specified.

## Conclusion

Our case reinforces the importance of rare, but potentially fatal, vascular injuries during cardiac catheterizations, such as TAVI. Furthermore, these vascular injuries need to be considered in the diagnostic process, especially if difficulties occur during the intervention. The importance of forensic investigations of cases similar to our case regarding quality control and prophylaxis have already been described decades previously [24]. However, because at this earlier time more extensive and potentially riskier interventions were not performed and the quantity of cardiac catheterizations was minimal, this issue needs to be emphasized again more stringently in the future.

The possibility of IAD should be considered and imaging of the aorta should be performed at an early stage if complications occur during the intervention. This case illustrates that even minor and initially irrelevant obstacles during TAVI may eventually lead to fatal complications, and therefore, they deserve recognition. Finally, unilateral pleural effusion post-intervention may be an indication of a developing hemothorax due to iatrogenic vessel injury.

## Key points

1. Even minor obstacles during TAVI may eventually lead to fatal complications.
2. Major vascular injuries need to be considered in the diagnostic process.
3. Imaging of the aorta should be performed at an early stage.
4. Unilateral pleural effusion post-intervention may indicate hemothorax.
